# Analgesic Effect of *Ziziphus abyssinica* Involves Inhibition of Inflammatory Mediators and Modulation of K_ATP_ Channels, Opioidergic and Nitrergic Pathways

**DOI:** 10.3389/fphar.2021.714722

**Published:** 2021-07-20

**Authors:** Isaac Tabiri Henneh, Francis Ackah Armah, Elvis Ofori Ameyaw, Robert Peter Biney, Ernest Obese, Eric Boakye-Gyasi, Emmanuel Awintiig Adakudugu, Martins Ekor

**Affiliations:** ^1^ School of Pharmacy and Pharmaceutical Sciences, University of Cape Coast, Cape Coast, Ghana; ^2^ Department of Biomedical Sciences, School of Allied Health Sciences, University of Cape Coast, Cape Coast, Ghana; ^3^ Department of Pharmacology, Faculty of Pharmacy and Pharmaceutical Sciences, Kwame Nkrumah University of Science and Technology, Kumasi, Ghana; ^4^ Department of Pharmacology, School of Medical Sciences, University of Cape Coast, Cape Coast, Ghana

**Keywords:** *Ziziphus abyssinica*, cytokines, inflammatory pain, anti-nociception, β-amyrin, polpunonic acid

## Abstract

The diversity offered by natural products has timelessly positioned them as a good source for novel therapeutics for the management of diverse medical conditions, including pain. This study evaluated hydro-ethanolic root bark extract of *Ziziphus abyssinica* (ZAE) as well as β-amyrin and polpunonic acid isolated from the plant for analgesic property. The study also investigated the mechanism responsible for this action in the extract. The antinociceptive potential of ZAE (30, 100, and 300 mg/kg, *p. o*.) was assessed using the tail-immersion test (TIT), acetic acid-induced writhing test (AAT), and formalin test (FT). The extract’s effect on acute and chronic musculoskeletal pain was also assessed by administering carrageenan unilaterally into the rat gastrocnemius muscles and measuring pain at 12 h and 10 days for acute and chronic pain respectively. The involvement of pro-inflammatory mediators (prostaglandin E_2_, bradykinin, TNF-α, and IL-1β) was assessed. The possible pathways mediating the observed analgesic effect of ZAE were further assessed using the antagonists: naloxone, glibenclamide, N^G^-L-nitro-arginine methyl ester (L-NAME), atropine, nifedipine, and yohimbine in the FT. Also the analgesic effect of two triterpenoid compounds, β-amyrin and polpunonic acid, previously isolated from the plant was assessed using the TIT. The anti-nociceptive activity of ZAE was demonstrated in the TIT by the significant (*p* < 0.05) increase in tail withdrawal threshold in ZAE-treated mice. ZAE also markedly reduced writhing and paw licking responses in both AAT and FT and significantly (*p* < 0.05) attenuated both acute and chronic musculoskeletal pain. ZAE also significantly reversed hyperalgesia induced by intraplantar injection of PGE_2_, bradykinin, TNF-α, and IL-1β. Furthermore, data revealed the involvement of opioidergic, ATP-sensitive K^+^ channels and NO-cGMP pathways in the analgesic effect of ZAE. Both β-amyrin and polpunonic acid exhibited analgesic activity in the tail suspension test. Our study demonstrates ZAE as an important source of new therapeutic agents for pain management.

## Introduction

One cardinal sign of inflammation is pain. The revised definition of pain by the International Society for the Study of Pain states that pain is “an unpleasant sensory and emotional experience associated with, or resembling that associated with, actual or potential tissue damage” (Raja et al., 2020). It is one of the major reasons people seek medical attention and accounts for most cases of absenteeism, unemployment, and underperformance at the workplace (Corrigan et al., 2011). Current modalities for pharmacological treatment of different types of pain are often not only inadequate but also pose several side effects to users such that recovery in most cases is impaired (Gilron, 2016). The search, therefore, for novel small molecules with therapeutic benefit, tolerable adverse effects, and wide margins of safety remains relevant for effective management of both acute and chronic pain.

In the past decades, herbal medicine has attracted worldwide attention as a versatile source of pharmacological agents with fewer adverse effects (Hou and Ng, 2014). The root bark of *Ziziphus abyssinica* is one of the medicinal plants that is widely used in folkloric medicine to treat various types of pain in many African countries (Burkil, 1985). Recently, we reported the anti-inflammatory property of the root bark extract of the plant (Henneh et al., 2018) as well as its protective effect against multi-organ toxicity induced by phenylhydrazine (Henneh et al., 2021). We have also isolated two triterpenes, beta-amyrin, and polpunonic acid, from the plant which showed prominent anti-arthritic potential (Henneh et al., 2020). Although we have reported some analgesic properties of the leaf extract of the plant (Boakye-Gyasi et al., 2017a, Boakye-Gyasi et al., 2017b), most of the plant’s medicinal properties have been ascribed to the roots. As such, further investigations into the root bark extract is still relevant since several studies have demonstrated that different parts of a plant can exhibit different or varying degrees of pharmacological effects (Al-Khazraji et al., 1993; Darabpour et al., 2011). Additionally, this current study seeks to investigate the effect of *Ziziphus abyssinica* root extract in acute and chronic musculoskeletal pain, which has been a major burden, particularly in the aged population (Blyth et al., 2019). The present study also investigates the analgesic potential of two compounds isolated from the root bark of the plant. The relevance of this current study is enhanced by the fact that the gastric ulcer, which limits the use of current analgesics, has been reported to be relieved by root extracts from the plant (Ugwah et al., 2013; Yau et al., 2017).

## Materials and Methods

### Plant Collection and Extraction

The collection of the fresh roots of *Ziziphus abyssinica* and the subsequent extraction with 70% ^v^/_v_ ethanol to obtained the extract (ZAE) has been described earlier (Henneh et al., 2018). Briefly, fresh root barks of *Ziziphus abyssinica* were collected from Ejura (7°23ʹ00.16ʺN, 1°22ʹ00.00ʺW) in the Ashanti Region of Ghana in the month of November 2016. It was authenticated at the Herbarium Unit of the Faculty of Pharmacy of the Kwame Nkrumah University of Science and Technology (KNUST) using a voucher specimen (KNUST/HM/2016/R003) which had been previously deposited at the herbarium. The name of the plant was verified from www.theplantlist.com (http://www.theplantlist.org/tpl1.1/record/kew-2471070) on 30th November 2017. The fresh root barks of *Ziziphus abyssinica* were air dried at room temperature for three weeks and pulverized into fine powder with the aid of a hammer mill. A portion (800 g) of the powdered roots was extracted with 5 L of 70% ^v^/_v_ ethanol for a 48 h period using Soxhlet Extraction Apparatus (Aldrich®, St. Louis, MO, United States). The extract obtained was labeled as ZAE and subsequently concentrated using a rotary evaporator (Rotavapor R-215 model, BÜCHI Labortechnik AG, Flawil, Switzerland) under reduced pressure and temperature (50°C). This was further dried on a water bath and then preserved in a desiccator containing activated silica until it was ready for use. The yield obtained was 8.7% ^w^/_w_.

**FIGURE 1:**
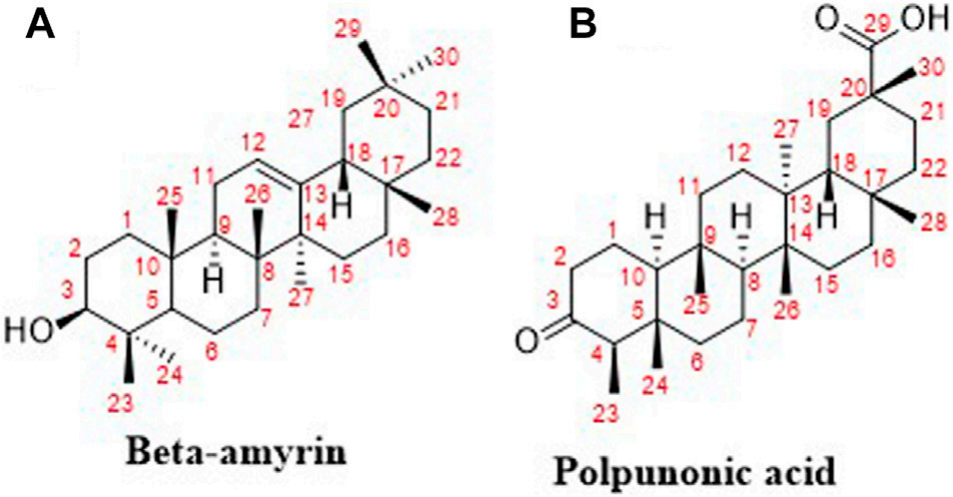
Chemical structures of isolated compounds from *Ziziphus abyssinica* (Henneh et al., 2020).

The two triterpenoid compounds, β-amyrin (Figure 1A) and polpunonic acid (Figure 1B) were obtained as fully described in our previous activity-guided isolation from the root bark of the plant (Henneh et al., 2020).

### Drugs and Chemicals

Formalin was purchased from BDH, Poole, England. Duopharma (M) Sdn Bhd, Malaysia, produced the naloxone hydrochloride, morphine sulfate, atropine sulfate used in the study. Nifedipine and diclofenac sodium were procured from Denk Pharma, Germany, whereas glibenclamide was manufactured by Sanofi-Aventis, Guildford, United Kingdom. We obtained yohimbine hydrochloride from Walter Ritter GmbH + Co. KG, Germany whiles prostaglandin E_2_, bradykinin acetate salt, murine recombinant tumor necrosis factor-alpha (TNF-α), and interleukin-1β, N^G^-L-nitro-arginine methyl ester (L-NAME) were purchased from the Sigma-Aldrich Inc. St. Louis, MO, United States.

### Animals

The study utilized male ICR mice (20–25 g) and Sprague–Dawley rats (170–250 g) procured from Noguchi Memorial Institute for Medical Research, Accra-Ghana. They were kept at the animal house facility of the Department of Biomedical Sciences, University of Coast, Ghana. in stainless steel cages (47 × 34 × 18) cm^3^ in groups of five rats or seven mice per cage. They were provided with unrestricted access to water and a standard rodent diet purchased from Flour Mills of Ghana, Tema, Ghana*.*

The protocols used in this study are in line with the “Principles of Laboratory Animal Care” (NIH Publication No. 85–23, Revised 1985) and established public health guidelines in Guide for Care and Use of Laboratory Animals (National Research Council, 2010). Ethical approval for the study was obtained from the University of Cape Coast Institutional Review Board (UCCIRB). ID: UCCIRB/CHAS/2016/13. University of Cape Coast, Cape Coast, Ghana.

### Acute Toxicity Test and Selection of Doses for *Ziziphus abyssinica*

To ensure that the doses of the extract used for the test did not produce any toxic or lethal effects, an acute toxicity test was performed in line with OECD (2001) guidelines. Thirty-five male Sprague-Dawley rats were randomly selected and divided into seven groups of five rats in each group. ZAE (30, 100, 300, 1,000, 3,000, and 5,000 mg/kg, *p. o*.) was orally administered to the respective group of animals. Control group rats received oral administration of distilled water (10 ml/kg). Animals were observed over 24 h after treatment for any changes in behavior or death. The rats were observed at 0, 15, 30, 60, 120, and 180 min, and 24 h after treatment for behaviors specifically related to central nervous system stimulation (hypersensitivity to external stimuli, excitation, stereotypies, jumping, aggressive behavior, and Straub tail), central nervous system depression (hyposensitivity to external stimuli, decreased muscle tone, loss of traction, hypothermia, loss of balance, sedation, motor incoordination, rolling gait, akinesia, and catalepsy) neurotoxicity (tremor and convulsions), and effects on autonomic functions, such as respiration, body temperature, salivation, urination, and defecation, were also noted. The rats were then observed daily for 14 days for any delayed toxicity.

Since no toxic signs or death were observed at doses as high as 5,000 mg/kg, the plant extract was deemed relatively safe. This paved the way for the assessment of the extract for its analgesic properties. With the toxicity data at hand, the doses of ZAE used in this study for its analgesic effect were selected based on preliminary studies in our laboratories as well as our earlier published data on the plant (Henneh et al., 2018; Henneh et al., 2021).

### Analgesic Tests

#### Tail–Immersion Test

The test was then same performed as previously described by Sewell and Spencer (1976) with modifications from Asante et al. (2019). This was done by immersing the lower 3.5 cm portion of the tail of each rat into a water bath (55 ± 0.5°C). The time taken for the rats to either flick or withdraw their tail was recorded as the latency to withdrawal. Baseline readings (pre-drug latency, L_1_) were taken before administration of ZAE (30, 100, and 300 mg/kg, p. o.), morphine (3 mg/kg, *p. o*.) or distilled water (10 ml/kg) and at 1, 2, 3, 4 and 5 h afterward (post-drug latency, L_2_). Control group rats received 10 ml/kg of distilled water. To prevent tissue damages, the cut-off latency (L_0_) for the test was set at 15 s. The percentage maximum possible effect (%MPE) was calculated using the formula below:

$$\%\mathrm{MPE}=\left(\frac{L_{2}-L_{1}}{L_{0}-L_{1}}\right)\times 100$$

#### Acetic Acid-Induced Writhing Test

A protocol initially described by Koster et al. (1959) and Ezeja *et al.* (2011) was adopted with some modifications. Five groups of mice (*n* = 5) were administered with either distilled water, three different doses of ZAE, or morphine as stated above under TIT. After 1 h, the mice were given 10 ml/kg of 0.6% ^v^/_v_ acetic acid via the intraperitoneal route. They were then separately placed in 20 testing Perspex chambers with dimensions 15 cm × 15 cm x 15 cm each. The nociceptive responses of the mice characterized by abdominal writhes were recorded for 30 min with a video camera and later traced using the JWatcher™ Version 1.0 software. Nociceptive score per 5 min time blocs was computed and analyzed.

#### Formalin Test in Mice

This test was carried out using an earlier established protocol (Hunskaar et al., 1985; Woode and Abotsi, 2011). Following acclimatization of the five groups (*n* = 5) of male ICR mice test conditions, they were treated with ZAE, morphine, or distilled water as stated earlier under TIT. One hour post-treatment, the mice were intraplantarly injected with 5% ^v^/_v_ formalin_,_ (10 µL/paw). Mice were transferred straightaway into the testing chambers and were their nociceptive behaviors (paw licking and biting) captured with the aid of a video camera for 1 h, tracked, and analyzed.

#### Effect of the Extract in Acute and Chronic Musculoskeletal Pain

##### Carrageenan-Induced Acute Musculoskeletal Pain

Acute muscle hyperalgesia was induced in rats by percutaneously injecting 0.1 ml of 3% carrageenan into their right gastrocnemius muscle (Radhakrishnan et al., 2003). Acute muscle pain develops within 12 h after injection, which was confirmed in all rats using the Randal-Selitto apparatus described earlier (Randall and Selitto, 1957). The weight that produced limb withdrawal or vocalization was recorded as the paw withdrawal latency. A cut-off weight of 250 g was used to avoid tissue damage. Baseline reading was taken before induction of hyperalgesia. Rats were then given various doses of ZAE, morphine, and normal saline as described earlier. The pain thresholds were measured again hourly for 5 h and the change in pain threshold resulting from the treatment of *Ziziphus abyssinica* and morphine was recorded. Paw withdrawal latencies were again measured hourly for 5 h after the various drug treatments to ascertain the effects of the various treatments on the pain threshold. Percentage maximum possible effect (%MPE) was calculated as follows:

$$\%\mathrm{MPE}=\frac{\left(\mathrm{postdrug treatment latency}-\mathrm{predrug treatment latency}\right)}{\left(\mathrm{Cut}\;\mathrm{off}\;\mathrm{latenc}y-\mathrm{predrug}\;\mathrm{treatment}\;\mathrm{latency}\right)}\times 100$$

##### Carrageenan Induced Chronic Musculoskeletal Pain

To measure the effect of ZAE on chronic musculoskeletal pain, the inflammation induced by the injected carrageenan was allowed to progress for ten days to allow the development of chronic pain as validated earlier (Radhakrishnan et al., 2003). Chronic hyperalgesia was confirmed in all rats by measuring the paw withdrawal latency of the right paw of the animals in the Randall-Selitto test. Paw withdrawal latencies before and after drug administration were measured as described in the acute protocol and the MPE computed accordingly.

### Mechanism of Action of the Extract

#### Tumor Necrosis Factor-Alpha (TNF-α) - Induced Hyperalgesia

Hyperalgesia was induced with intraplantar administration of TNF-α irritant (2.5 pg/paw; 20 µL) (Vale et al., 2004; Boakye-Gyasi, et al., 2017b) following 1-h pre-treatment of rats with various doses of ZAE or morphine to designated groups of rats (*n* = 5). Distilled water (10 ml/kg) was orally administered to the rats in the control group. Intraplantar injection of TNF-α into the right hind paw produced hyperalgesia which was measured at times 0, 1, 2, 3, 4, and 5 h using an analgesimeter (Ugo Basile, Comerio, Varese, Italy) as has been described previously (Randall and Selitto, 1957).% MPE was calculated as follows:

$$\%\mathrm{MPE}=\left(\frac{\mathrm{PWT}t-\mathrm{PWT}o}{250g-\mathrm{PWT}o}\right)\times 100$$

Where PWT*t* is the paw withdrawal threshold at time t and PWT*o* is the paw withdrawal threshold at time zero (0). The cut-off latency was set at 250 g.

#### Interleukin-1β -Induced Hyperalgesia

Rats were pre-treated with various doses of ZAE, morphine, or distilled water for 1 h before intraplantar injection with 20 µL of 1 pg IL-1β per right hind paw (Vale et al., 2004; Boakye-Gyasi et al., 2017). Hyperalgesia was measured in the injected paws at 1, 3, and 5 h post-IL-1β injection as described above.

#### Bradykinin-Induced Hyperalgesia

Rats were pretreated with ZAE, distilled water, or morphine for 1 h before intraplantar injection with 20 µL of 500 ng bradykinin per right hind paw. (Vale et al., 2004). Hyperalgesia was measured in the injected paws at 1, 3, and 5 h post bradykinin administration as described earlier. To avoid breakdown of the injected bradykinin by angiotensin-converting enzyme (ACE), rats were subcutaneously injected with 5 mg/kg captopril (an antagonist of the enzyme) 1 h prior to the experiment.

#### Prostaglandin E_2_ - Induced Hyperalgesia

Hyperalgesia was induced in rats with 20 µL of 100 ng of prostaglandin E_2_ (PGE_2_) irritant per right hind paw (Vale et al., 2004). One hour before PGE_2_ administration, the animals were treated with various doses of ZAE, morphine, or distilled water. The anti-nociceptive effect of the test agents was assessed as earlier described.

#### Involvement of Nociceptive Pathways

To investigate the involvement of various nociceptive pathways of nociception in the analgesic activity of the extract, various groups of mice (*n* = 5) were orally or intraperitoneally pretreated with antagonists such as naloxone, 2 mg/kg, i. p.; glibenclamide 8 mg/kg, p. o.; L-NAME 10 mg/kg i. p.; yohimbine 3 mg/kg, p. o.; atropine 3 mg/kg, i. p. or nifedipine10 mg/kg, p. o*.* Doses of the various antagonists, as well as morphine, were selected based on preliminary studies in our laboratories as well published literature (Hendrie, 1989; Woode and Abotsi, 2011; Boakye-Gyasi et al., 2017b; Asante et al., 2019; Obese et al., 2021). Thirty (p.o.) or fifteen (i.p.) min post-antagonists treatment, mice were given ZAE (100 mg/kg, p. o.). One hour after ZAE treatment, nociception was induced with 10 µL of 5% formalin in all groups, and the nociceptive score was measured for 1 h and analyzed as described above in the formalin test. The procedure was repeated for another set of six groups of mice that received morphine (3 mg/kg) instead of ZAE. Distilled water (10 ml/kg) was orally administered to the negative control group, while ZAE- and morphine-treated controls received only ZAE (100 mg/kg) or morphine (3 mg/kg) respectively.

### Analgesic Effect of the Isolated Compounds

The tail immersion test was adopted to test for the analgesic effect of the two isolated compounds from the plant, β-amyrin, and polpunonic acid at doses of 3, 10, and 30 mg/kg using the method described earlier. Morphine, a standard reference analgesic, was tested at oral doses of 1, 3, and 10 mg/kg whereas the negative control group received distilled water (10 ml/kg). The doses of β-amyrin and polpunonic acid used in the test were selected based on preliminary studies and our earlier published data on the compounds (Henneh et al., 2020).

### Statistical Analysis

Data in this research article have been presented as mean ± S.E.M. Graphs and statistical analyses have been performed with GraphPad® Prism version seven for Windows Version 10. The symbols *p < 0.05; **p < 0.01; ***p < 0.001 denote significant differences in time-course curves of treatment compared to the control (Ctrl) group (two-way ANOVA followed by Dunnet’s multiple comparison test) whereas ^†^ *p* < 0.05, ^††^ *p* < 0.01, ^†††^ *p* < 0.001 denote significant differences of treatment in comparison with control (Ctrl) group using the one-way ANOVA followed by Tukey’s multiple comparison test.

Nonlinear regression with three-parameter logistic analysis was employed in determining the dose-response curves as well as the doses of test agents (β-amyrin, polpunonic acid, and morphine) that produced 50% of the maximal effect (ED_50_).

## Results

### Analgesic Tests

#### Tail Immersion Test

**FIGURE 2:**
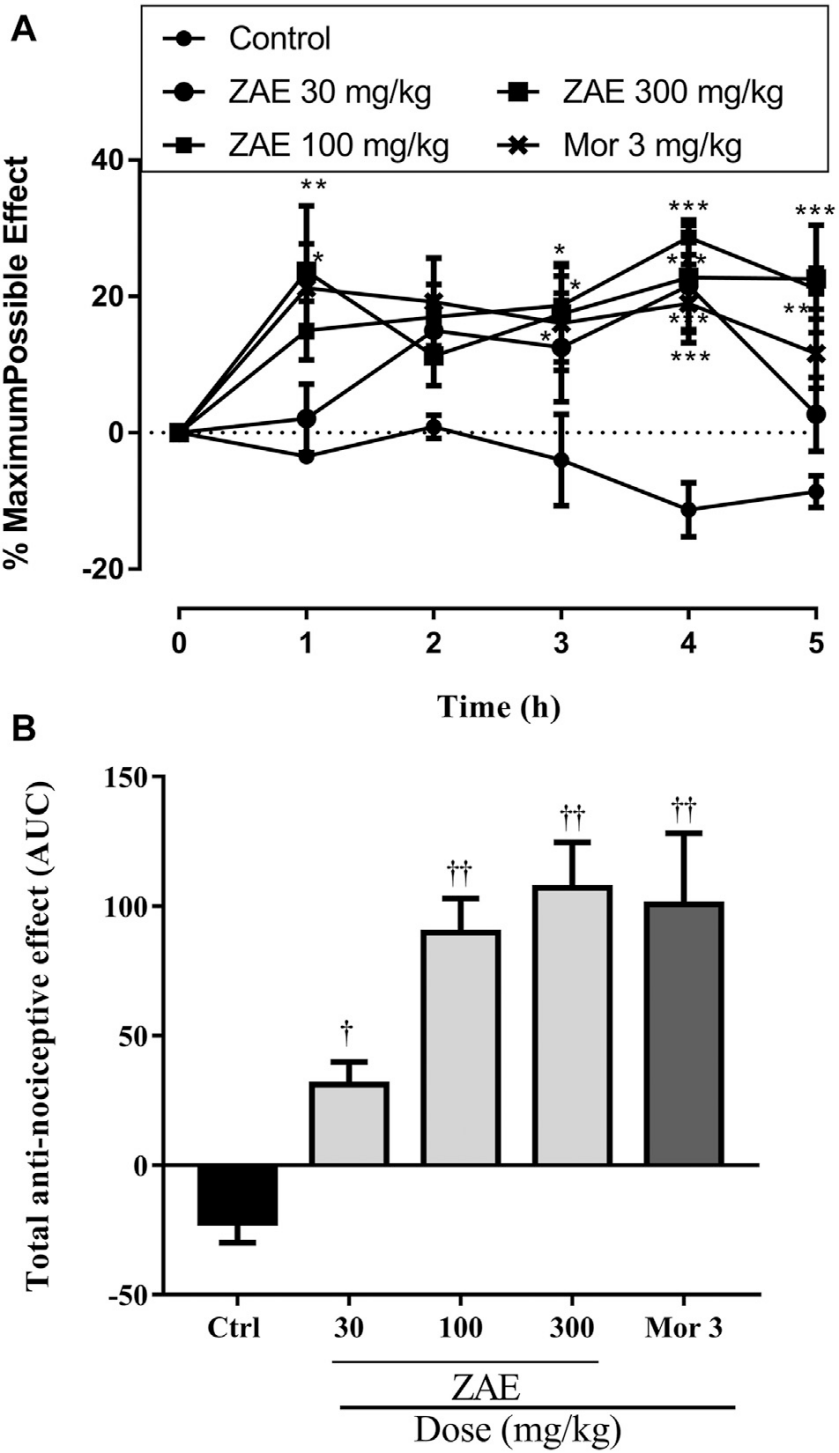
The total anti-nociceptive effect of three doses of ZAE and morphine presented **(A)** time-course curves of the various treatments over 5 h and **(B)** areas under the curve (AUC) in tail-immersion test in mice. The symbols *p < 0.05; **p < 0.01; ***p < 0.001 denote significant differences in time-course curves of treatment compared to the control (Ctrl) group (two-way ANOVA followed by Dunnet’s multiple comparison test) whereas ^†^ *p* < 0.05, ^††^ *p* < 0.01, ^†††^ *p* < 0.001 denote significant differences of treatment in comparison with control (Ctrl) group using the one-way ANOVA followed by Tukey’s multiple comparison test.

All three dose levels of ZAE (30, 100, and 300 mg/kg, p. o.) showed significant (*p* < 0.01) inhibition compared to the control (vehicle). The positive control, morphine (3 mg/kg, p. o.), also demonstrated significant inhibition compared to the negative control as shown in Figure 2.

#### Acetic-Acid Induced Nociception

**FIGURE 3:**
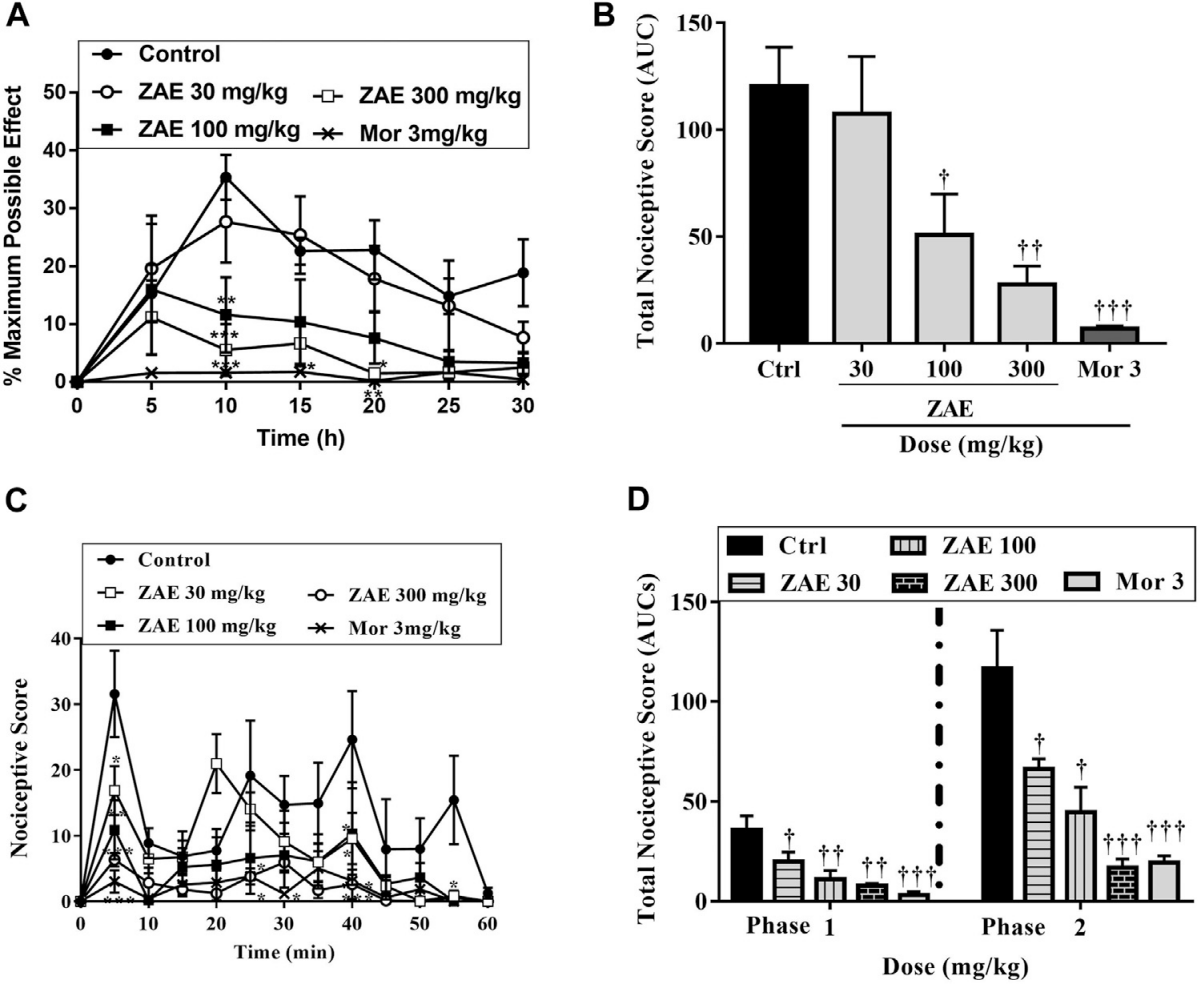
Effect of the three doses of ZAE and morphine on the **(A)** percentage maximum response **(B)** total nociceptive score of the acetic acid-induced writhing test presented as the area under the curve (AUC). The figure also presents the effect of the three doses of ZAE and morphine on the **(C)** percentage maximum response and **(D)** total nociceptive score of the formalin test presented as the area under the curve (AUC). The symbols *p < 0.05; **p < 0.01; ***p < 0.001 denote significant differences in time-course curves of treatment compared to the control (Ctrl) group (two-way ANOVA followed by Dunnet’s multiple comparison test) whereas ^†^ *p* < 0.05, ^††^ *p* < 0.01, ^†††^ *p* < 0.001 denote significant differences of treatment in comparison with control (Ctrl) group using the one-way ANOVA followed by Tukey’s multiple comparison test.

Administration of ZAE significantly (*p* < 0.001; Figure 3A,B reduced the nociceptive effect of acetic acid. ZAE (30, 100, and 300 mg/kg) treatment resulted in a mean percentage inhibition of nociception by 10.68, 57.82, and 76.94%, respectively. Morphine inhibited the writhing response by 94.21%.

#### Formalin Test

Results presented in Figure 3C,D show that the administration of formalin into the paw of the mice produced a typical biphasic nociceptive response consisting of an initial, rapid-licking acute neurogenic phase (phase I) within 10 min, followed by a slowly rising and long-lived 10–60 min inflammatory phase (phase II). In the neurogenic phase, administration of 30, 100, and 300 mg/kg of the extract to the mice produced an ameliorative effect with a mean percentage inhibition of 44.11, 69.50, and 78.43% respectively with morphine (3 mg/kg) producing 85.48%. In the inflammatory phase, ZAE 30, 100, and 300 mg/kg reversed formalin-induced nociception with mean percentage inhibition of 43.16%, 61.82%, and 83.36.96%. This was comparable to the effect of morphine (3 mg/kg) which produced a percentage inhibition of 90.96%.

#### Acute and Chronic Musculoskeletal Pain

**FIGURE 4:**
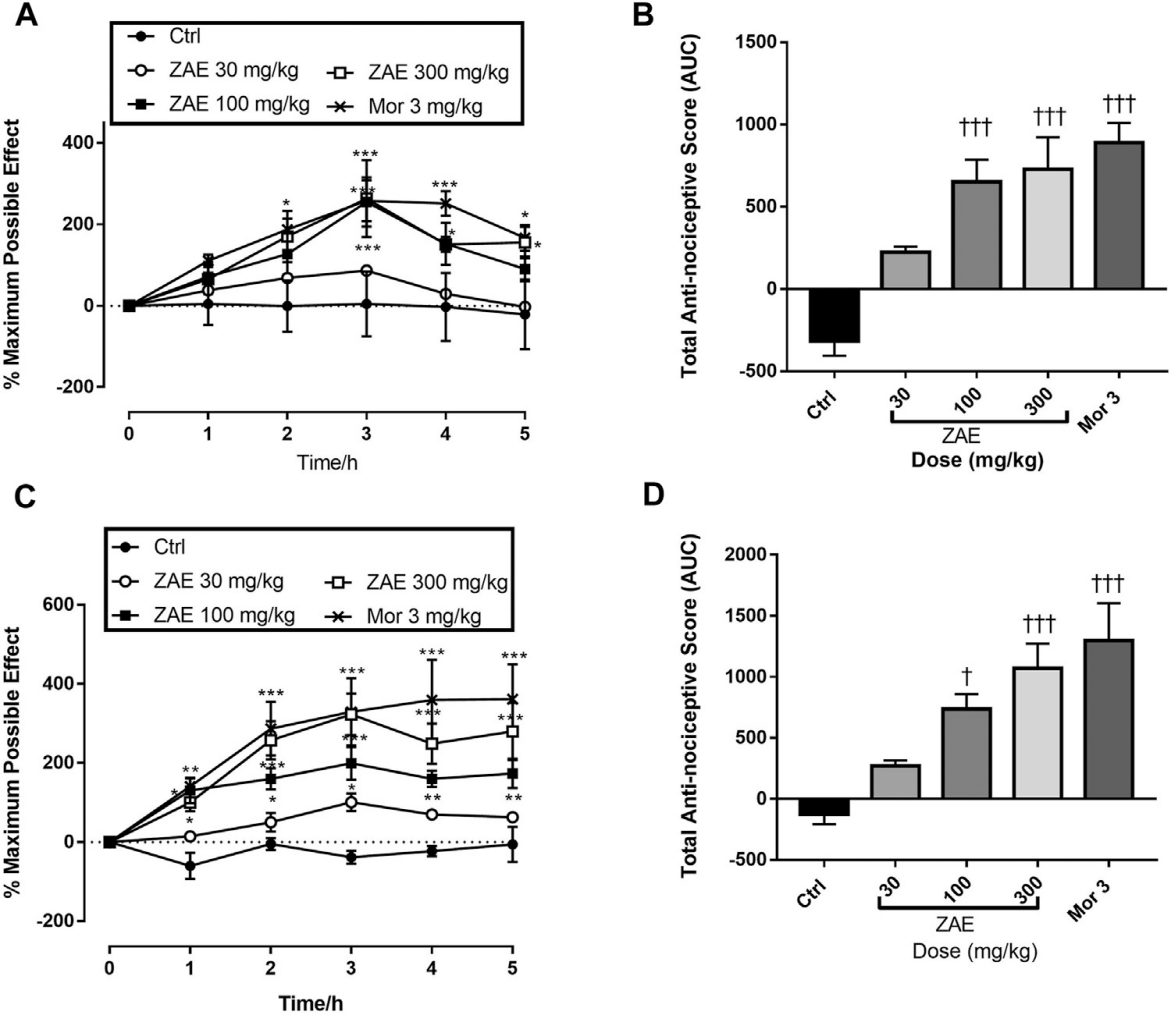
Effect of the three doses of ZAE and morphine on the **(A)** percentage maximum possible effect and **(B)** total anti-nociceptive effect in acute musculoskeletal pain in rats. The figure also shows the effect of the three doses of ZAE and morphine on the **(C)** percentage maximum possible effect and **(D)** total anti-nociceptive effect in chronic musculoskeletal pain in rats. The symbols *p < 0.05; **p < 0.01; ***p < 0.001 denote significant differences in time-course curves of treatment compared to the control (Ctrl) group (two-way ANOVA followed by Dunnet’s multiple comparison test) whereas ^†^ *p* < 0.05, ^††^ *p* < 0.01, ^†††^ *p* < 0.001 denote significant differences of treatment in comparison with control (Ctrl) group using the one-way ANOVA followed by Tukey’s multiple comparison test.

Figure 4A shows the time-course curves of the ZAE, morphine, and vehicle-treated groups following carrageenan-induced acute musculoskeletal pain in rats. The column graphs of vehicle control, ZAE, and group morphine are presented in Figure 4B. *Ziziphus abyssinica* (30, 100, and 300 mg/kg, p. o) significantly and dose-dependently inhibited carrageenan-induced acute muscle hyperalgesia in the time-course curve (*p* < 0.05) in the ipsilateral limb compared to the normal saline-treated group. Morphine (3 mg/kg, i. p) used as a positive control also significantly (*p* < 0.001) inhibited acute muscle hyperalgesia over the 5 h of pain measurement.

Similarly, there was a significant and dose-related increase in the total antinociceptive effect of ZAE (100 (*p* < 0.05) and 300 mg/kg (*p* < 0.01) respectively in carrageenan-induced chronic muscle pain in rats as can be observed in Figure 4C,D. This was comparable to the effect produced by morphine at 3 mg/kg.

### Mechanisms of Action of the Extract

#### TNF-α and Interleukin-1β - Induced Hyperalgesia

**FIGURE 5:**
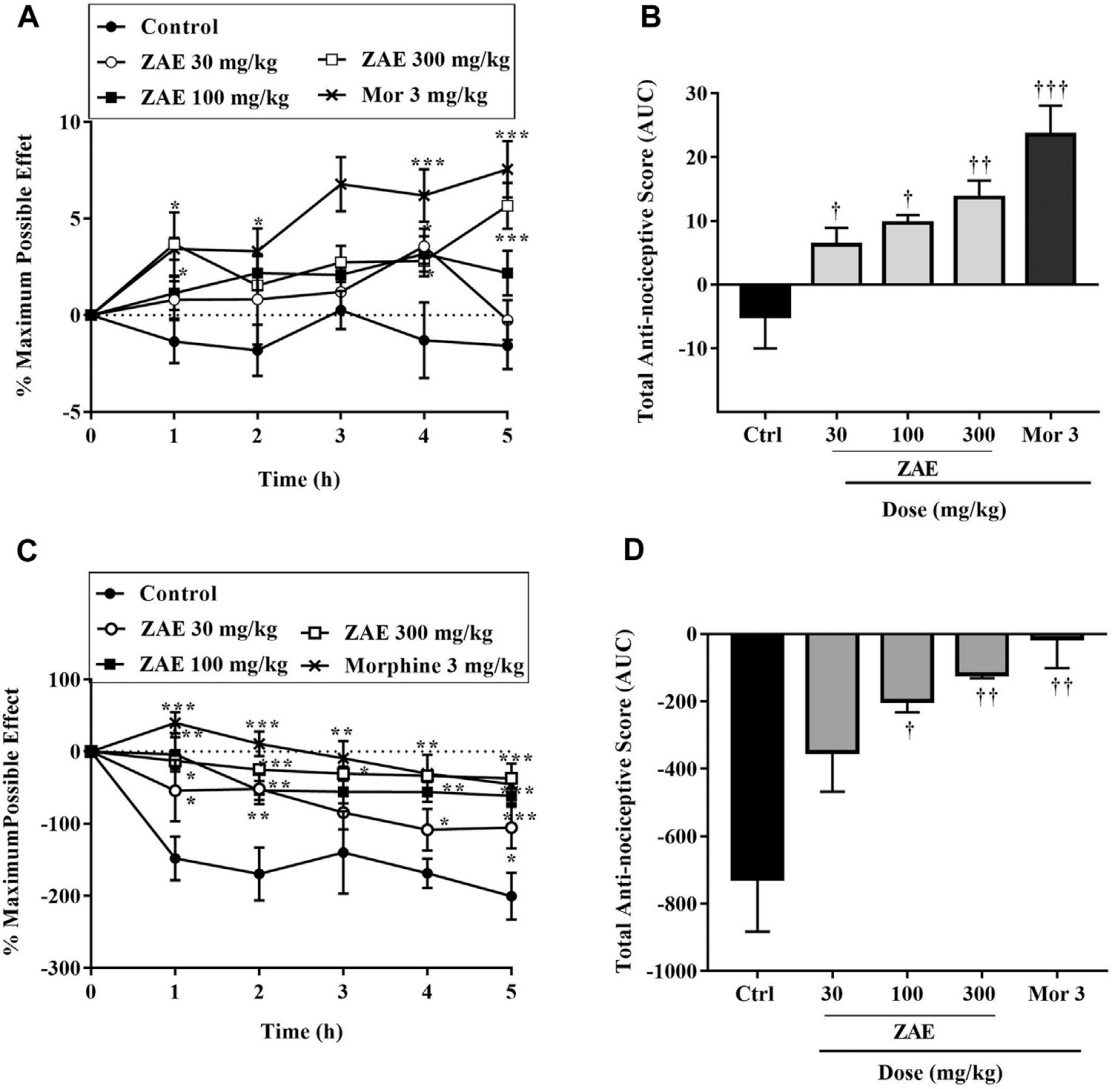
Effect of pre-treatment of rats with ZAE and morphine on TNF-α **(A, B)** and IL-1β **(C, D)** - induced hyperalgesia. Data **(A and C)** represent the time-course curves whereas **(B and D)** represent total anti-nociceptive effects (AUC). The symbols *p < 0.05; **p < 0.01; ***p < 0.001 denote significant differences in time-course curves of treatment compared to the control (Ctrl) group (two-way ANOVA followed by Dunnet’s multiple comparison test) whereas ^†^ *p* < 0.05, ^††^ *p* < 0.01, ^†††^ *p* < 0.001 denote significant differences of treatment in comparison with control (Ctrl) group using the one-way ANOVA followed by Tukey’s multiple comparison test.

Mechanical hyperalgesia produced following TNF-α injection was significantly (*p* < 0.001) reversed by ZAE as well as morphine (3 mg/kg) (Figure 5A,B. Similarly, intraplantar administration of IL-1β induced a nocifensive response in rats, and this was significantly (*p* < 0.01) reduced by ZAE 100 and 300 mg/kg as well as morphine 3 mg/kg (Figure 5C,D).

#### Bradykinin and Prostaglandin E_2_-Induced Hyperalgesia

**FIGURE 6:**
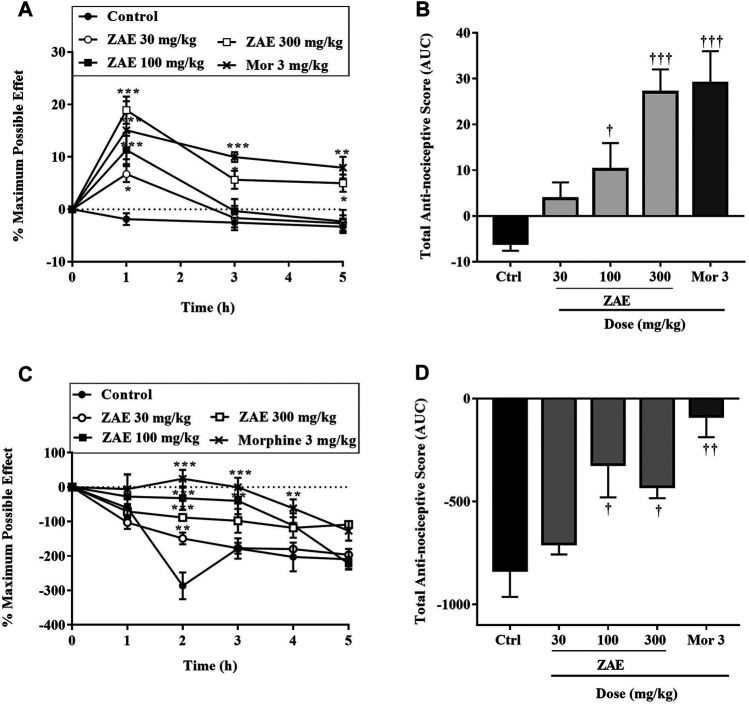
Effect of pre-treatment of rats with ZAE and morphine on bradykinin **(A, B)** and prostaglandin E_2_ (C and D) - induced hyperalgesia. Data (C and D) represent the time-course curves whereas **(B–D)** represent total anti-nociceptive effects (AUC). The symbols *p < 0.05; **p < 0.01; ***p < 0.001 denote significant differences in time-course curves of treatment compared to the control (Ctrl) group (two-way ANOVA followed by Dunnet’s multiple comparison test) whereas ^†^ *p* < 0.05, ^††^ *p* < 0.01, ^†††^ *p* < 0.001 denote significant differences of treatment in comparison with control (Ctrl) group using the one-way ANOVA followed by Tukey’s multiple comparison test.

The administration of the extract of *Ziziphus abysinnica* significantly (*p* < 0.001; Figure 6A,B reversed the hyperalgesia in rats that received ZAE. Morphine (also significantly reversed the hyperalgesia comparable to that of ZAE 300 mg/kg. Also, prostaglandin E_2_ irritant-induced hyperalgesia was significantly (*p* < 0.01) reversed by oral administration of 100 and 300 mg/kg of ZAE and morphine 3 mg/kg (Figure 6C,D).

#### Assessment of the Involvement of Nociceptive Pathways Using the Formalin Test

**FIGURE 7:**
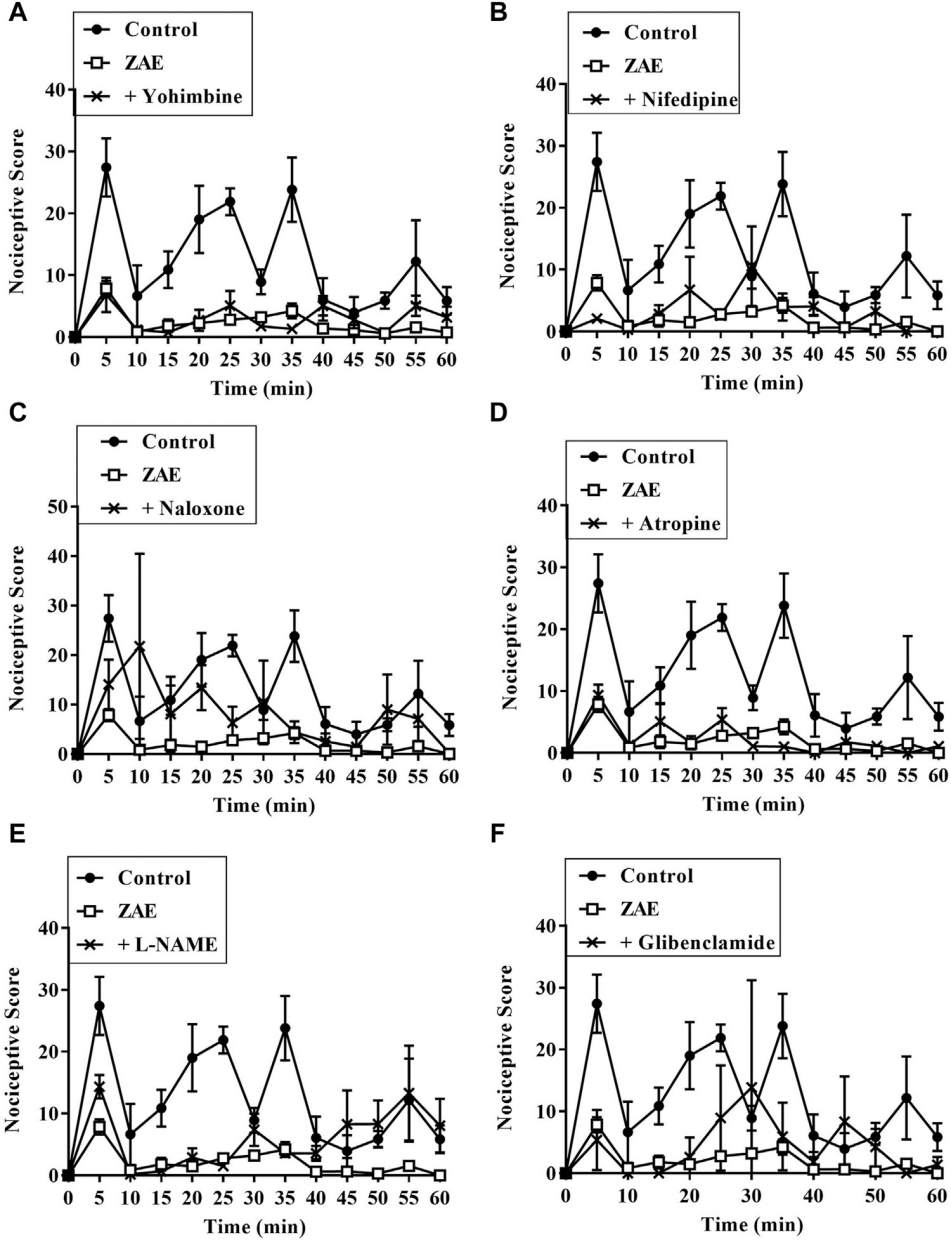
Effect of pre-treatment of mice with **(A)** yohimbine **(B)** nifedipine **(C)** naloxone **(D)** atropine **(E)** L- NAME, and **(F)** glibenclamide on the anti-nociceptive profile of ZAE (100 mg/kg, p. o.) in phase 1 and phase 2 of formalin-induced nociception.

**FIGURE 8:**
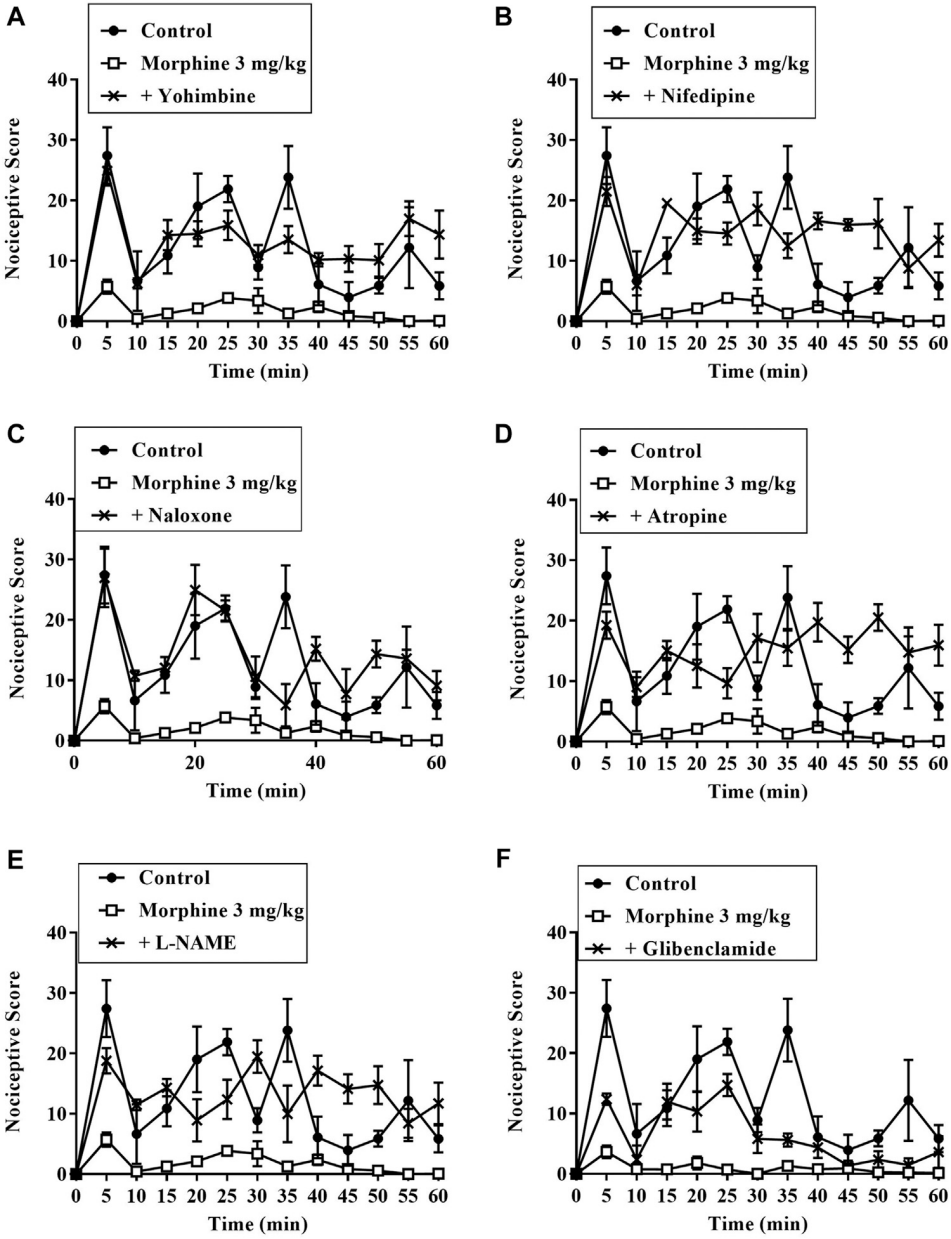
Effect of pre-treatment of mice with **(A)** yohimbine **(B)** nifedipine **(C)** naloxone **(D)** atropine **(E)** L- NAME, and **(F)** glibenclamide on the anti-nociceptive profile of morphine (3 mg/kg, p. o.) in phase 1 and phase 2 of formalin-induced nociception.

**FIGURE 9:**
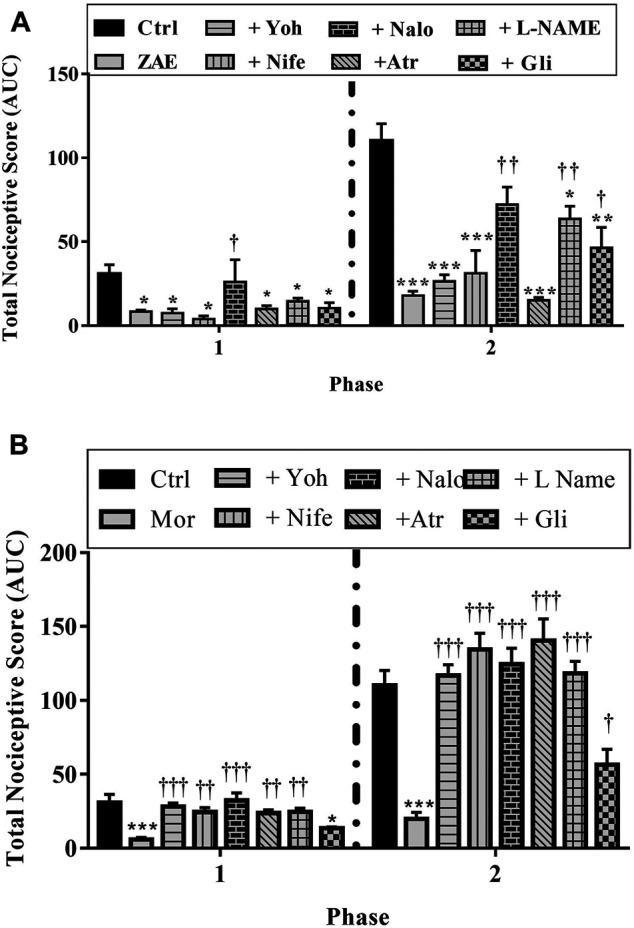
Effect of pre-treatment of mice with yohimbine, nifedipine, naloxone, atropine, L- NAME, and glibenclamide on the anti-nociceptive profile of **(A)** ZAE (300 mg/kg, p. o.) and **(B)** morphine (3 mg/kg, i. p.) in phase 1 and phase 2 of formalin-induced nociception. Each column represents the mean of five animals and the error bars indicate S.E.M. ***p < 0.001 compared to respective vehicle-treated controls; ^†††^ *p <* 0.001, ^††^ *p <* 0.01 and ^†^ *p <* 0.05 compared to either ZAE 100 mg/kg or morphine 3 mg/kg (all one-way ANOVA followed by Tukey’s post *hoc* test).

Results presented in Figures 7–9 show potential pathways mediating the analgesic activities of ZAE and morphine. The analgesic effect of ZAE was significantly reversed in both phases of the formalin test by the pretreatment of mice with naloxone, whereas L-NAME and glibenclamide reversed only the inflammatory phase of the formalin test. The analgesic effect of morphine, on the other hand, was significantly diminished by yohimbine, nifedipine, naloxone, atropine, and L-NAME. Glibenclamide, however, significantly reversed the pain in the inflammatory but not the neurogenic phase of the test.

#### Analgesic Effect of the Isolated Compounds

**FIGURE 10:**
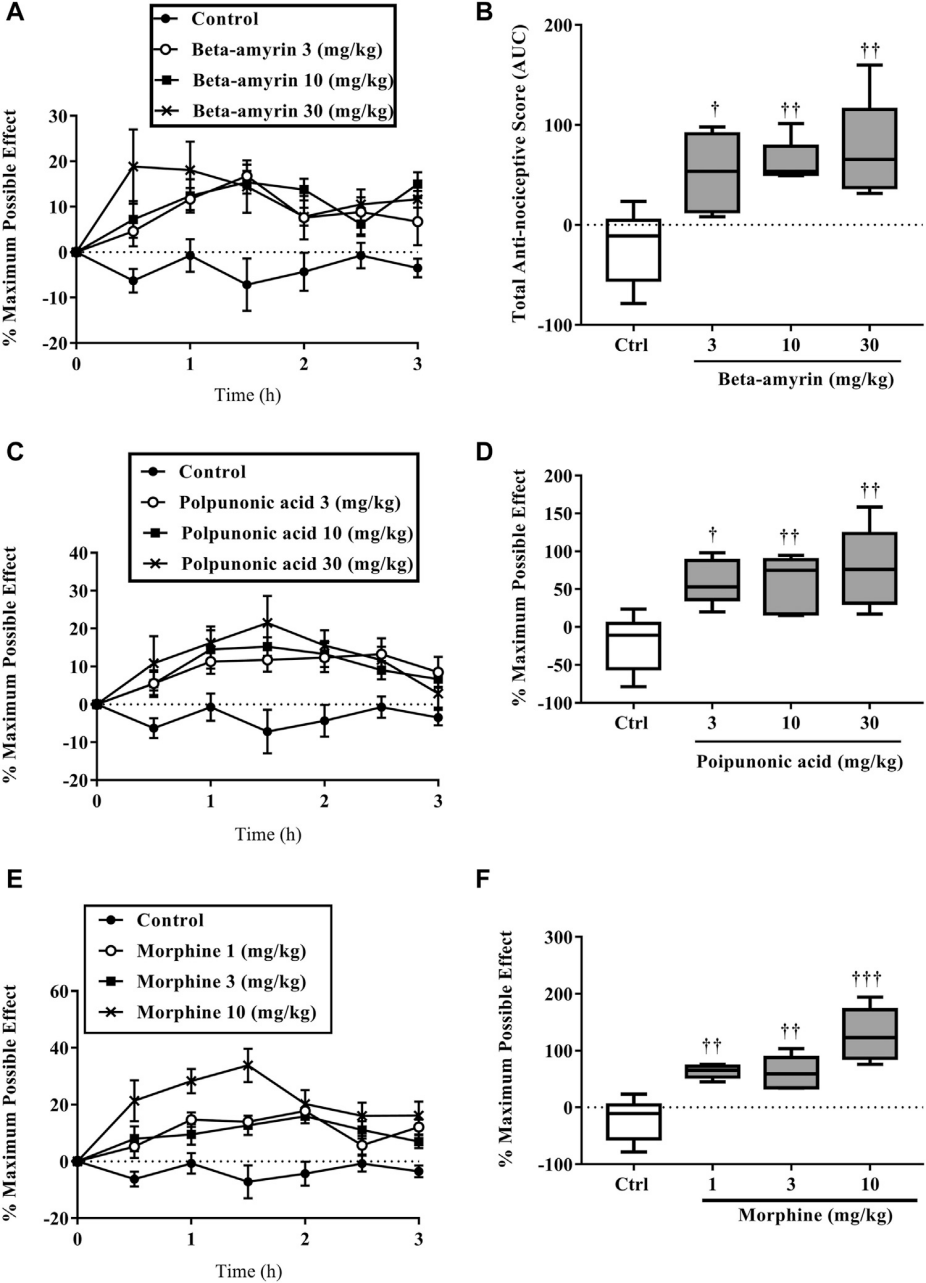
The percentage maximum possible effect **(A, C, D)** and total antinociceptive effect **(B, D, E)** of β-amyrin (3, 10 and 30 mg/kg, p. o.), polpunonic acid (3, 10 and 30 mg/kg, p. o) and morphine (1, 3 and 10 mg/kg, p. o) presented as the area under the curve (AUC) in tail-immersion test in mice. The symbols *p < 0.05; **p < 0.01; ***p < 0.001 denote significant differences in time-course curves of treatment compared to the control (Ctrl) group (two-way ANOVA followed by Dunnet’s multiple comparison test) whereas ^†^ *p* < 0.05, ^††^ *p* < 0.01, ^†††^ *p* < 0.001 denote significant differences of treatment in comparison with control (Ctrl) group using the one-way ANOVA followed by Tukey’s multiple comparison test.

**FIGURE 11:**
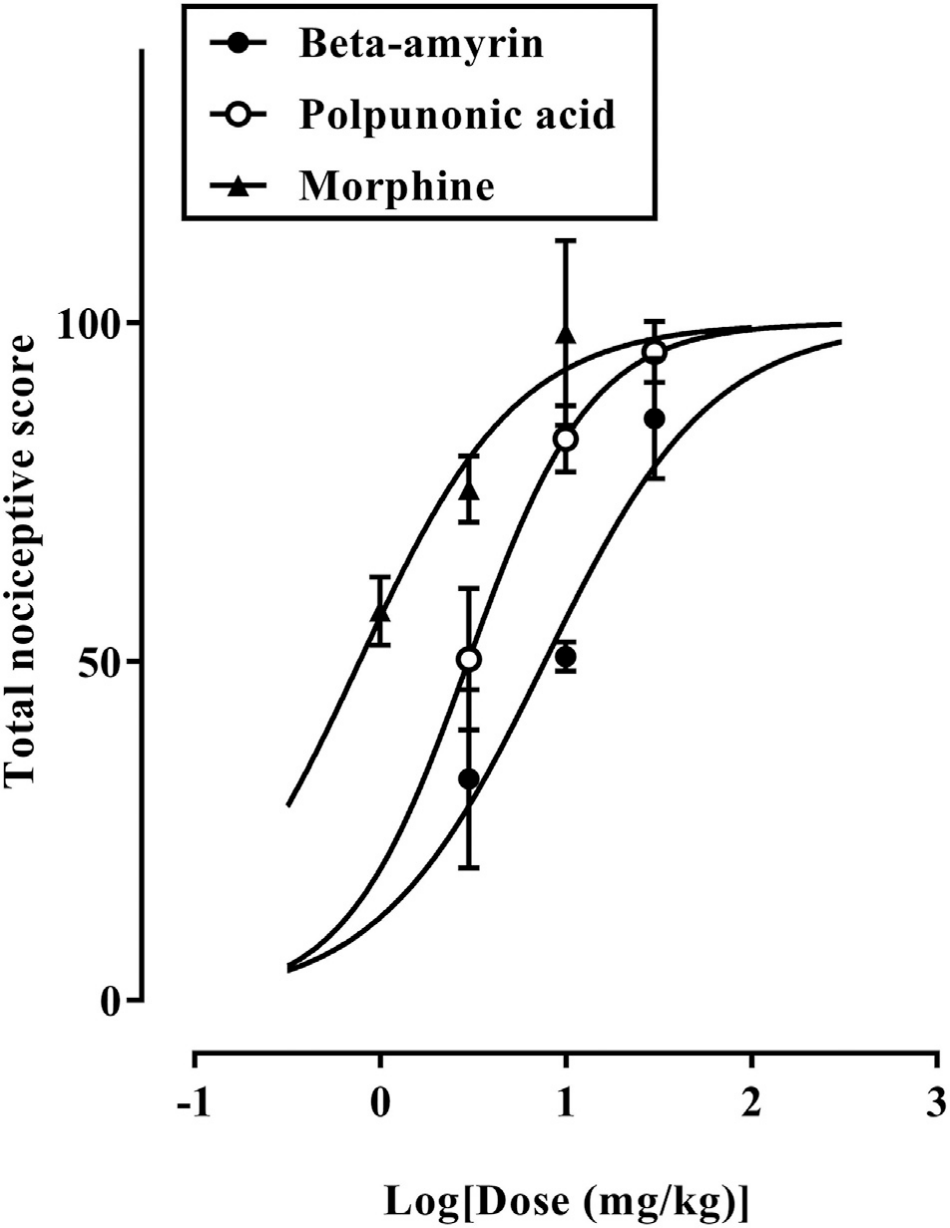
Dose-response curves of β-amyrin, polpunonic acid, and morphine.

β-amyrin, polpunonic acid, and morphine at all tested doses exhibited significant anti-nociceptive effects. Dose-response curves plotted revealed that morphine was the most potent, followed by polpunonic acid and β-amyrin with ED_50s_ of 0.92, 3.70, and 5.51 mg/kg (Figures 10, 11).

## Discussion

The present study provides evidence for the analgesic property of the root bark extract and isolated bioactive compounds (β-amyrin and polpunonic acid) from *Ziziphus abyssinica* and the mechanism(s) by which this effect is produced.

To do this, the tail immersion test was employed in assessing the central analgesic effect of the extract. The test utilizes thermal stimuli between 50 and 55°C to evaluate the central anti-nociceptive activity of medicinal agents. An agent’s ability to increase the reaction time of rodents in this model is considered as an essential index for central analgesic effects (Abdelhalim et al., 2015) and this was observed in ZAE-treated animals. Other researchers have correlated this property of medicinal agents with central anti-nociceptive mechanisms through the activation of opioidergic receptors (Hasan et al., 2018). Specifically, this thermal stimulus affects the μ2/δ and the μ1/μ2-opioid receptors which mediate spinal and supraspinal reflex mechanisms, respectively (Turner, 2013; Moniruzzaman and Imam, 2014).

Due to the multidimensional nature of pain, it was expedient to further explore the effect of the extract in other models. As such, the acetic-acid induced writhing test was used to further evaluate the analgesic effect of ZAE. This model is known to implicate both visceral and inflammatory pain (Agarwal and Kansal, 2018). Intraperitoneal injection of acetic acid is known to activate pain sensation through a localized inflammatory response leading to the release of free arachidonic acid from tissue phospholipids via the activity of cyclooxygenase leading to increased production of prostaglandins (PGE_2_ and PGF_2_) in peritoneal fluids (Uddin et al., 2018). Other related studies have linked IL-1β, IL-8, and tumor necrosis factor-alpha (TNF-α) from mast cells and resident macrophages within the peritoneum as contributing to the nociception caused by acetic acid (Ribeiro et al., 2000). ZAE significantly decreased the writhing response induced by intraperitoneal injection of acetic acid, and this is suggestive of its analgesic effect possibly through the inhibition or downregulation of inflammatory mediators and cytokines such as IL-1β, IL-8, and tumor necrosis factor-alpha (TNF-α) from mast cells and resident macrophages within the peritoneum.

Although the acetic-acid induced writhing test is simple, sensitive, and particularly suitable for detecting even weaker analgesics, it is not a selective pain test as it gives false-positive results for some non-analgesics such as muscle relaxants and sedatives (Le Bars et al., 2001). Therefore, to further confirm the analgesic action of ZAE, the formalin test was used. This test is beneficial for the assessment of novel analgesic drugs due to its highly predictive nature and its ability to mimic both acute and chronic pain. It also encompasses neurogenic, inflammatory, and central mechanisms of nociception (Tjølsen et al., 1992; Le Bars et al., 2001). The biphasic response of the formalin test involves neurogenic (0–10 min) and inflammatory (10–60 min) phases and drugs such as morphine is known to inhibit both stages.

In this study, ZAE inhibited both phases of the test similar to morphine. The neurogenic (early) phase is a result of direct stimulation of nerve endings leading to the release of substance P and bradykinin through central mechanisms. The late phase, also termed the inflammatory phase, is mediated by peripheral effects through the release of some inflammatory mediators such as histamine, serotonin, bradykinin, and prostaglandins which to some extent can also cause sensitization of central nociceptive neurons (Uddin et al., 2018). The inhibitory effect of ZAE in both phases indicates that the extract had central as well as peripheral analgesic properties. It also confirms the central analgesic properties observed in the tail immersion test. Furthermore, the inhibition of the inflammatory phase by the extract is not surprising as it confirms our earlier report which revealed that ZAE possesses anti-inflammatory activity (Henneh et al., 2018).

Aside from nociceptive and inflammatory pain, musculoskeletal pain also underlines several pathological conditions and constitutes a substantial disease and economic burden in many individuals (Nielsen and Henriksson, 2007). As such, agents which are effective in several types of pain may help reduce polypharmacy, with its attendant drug interactions and adverse effects. It was therefore expedient to explore the effect of ZAE in both acute and chronic musculoskeletal pain. Oral administration of hydro-ethanolic root bark extract of *Ziziphus abyssinica* exhibited analgesic properties by ameliorating acute and chronic muscle pain induced by 3% carrageenan in rats. The type of musculoskeletal pain induced in this study is known to be closely related to fibromyalgia, which is a generalized type of muscle pain experienced in humans (Skyba et al., 2005). In this test, secondary hyperalgesia leading to chronic musculoskeletal pain has been shown to occur as a result of central sensitization emanating from the activation of dorsal horn neurons, together with the increased sensitivity of the peripheral nociceptors (Sluka and Westlund, 1993). Both acute and chronic hyperalgesia were ameliorated significantly by the extract. This could be attributed to the extract’s effect on the inhibition of neutrophil degranulation as reported earlier (Henneh et al., 2018), as well as its effect on prostaglandins and other inflammatory mediators reported in this study.

Again, ZAE exhibited analgesic activity in the acetic acid, formalin as well as acute and chronic musculoskeletal pain models and for the fact that all these models mediate pain through inflammatory mediators, we investigated the effect of the extract in the presence of some of these mediators. It was observed in those experiments that ZAE reversed hyperalgesia induced by the intraplantar injection of TNF-α, IL-1β, prostaglandin E_2_ and bradykinin in rats. This pathway follows an earlier hypothesis that intraplantar injection of TNF- α irritant triggers IL-1β production, thus inducing the production of cyclooxygenase products such as prostaglandin E_2_ which subsequently causes hyperalgesia through sensitization of nociceptors by bradykinin and serotonin (Verri Jr et al., 2007). This was supported by the ability of ZAE to inhibit hyperalgesia induced by TNF-α and other downstream mediators, IL-1β, prostaglandin E_2,_ and bradykinin. The analgesic effect of the extract in both acetic acid-induced writhing and formalin-induced nociception in mice as reported in this study could therefore be attributed to its inhibitory effect on these inflammatory mediators and cytokines.

To appreciate the mode of analgesic activity of the extract, further investigations were conducted using the formalin test. The results revealed that the analgesic effect of ZAE was reversed by pretreatment of animals with naloxone, an opioid antagonist, in both phases of the formalin test. Previous studies have implicated the opioidergic pathway in the analgesic effect of the leaf extract of *Ziziphus abyssinica* (Boakye-Gyasi et al., 2017b) as well as other plant extracts (Al-Afifi et al., 2018; Asante et al., 2019)*.* ZAE could, therefore, be said to mediate its analgesic effect either through the activation of opioidergic receptors or upregulation of endogenous opioidergic agonists. This is supported by the extract’s ability to inhibit thermal nociception, which is also known to be mediated by μ2/δ -opioid receptors as well as μ1/μ2-opioid receptors (Turner, 2013; Moniruzzaman and Imam, 2014).

Results obtained from this study further reveal that the extract could possibly also be mediating its analgesic activity via the nitric oxide pathway which is modulated by the μ-opioid receptors. The μ-opioid receptor is coupled with nitric oxide synthase (NOS) which regulates the production of nitric oxide. NO’s role in analgesia involves the downregulation of neuronal pain transmission by obstructing Ca^2+^ influx whiles activating K^+^ channels opening. These lead to hyperpolarization and subsequent inhibition of action potentials generation, thus curtailing the transmission of nociceptive signals peripherally and centrally (Stefano and Kream, 2007; Cury et al., 2011; Galdino et al., 2015; Gomes et al., 2020). The extract, therefore, may be involved in a cascade of events (particularly via the opioidergic pathway) that possibly led to the activation of nitric oxide synthase, an enzyme required for the production of pain-relieving nitric oxide.

Furthermore, results from this study showed that ZAE possibly mediates its activity through the activation of ATP-sensitive potassium channels. This activation of the channel causes efflux of potassium from post-synaptic neurons and a subsequent hyperpolarization. This happens following the activation of post-synaptic activation of opioidergic receptors. It is worth noting that a series of investigations have highlighted a marked implication of the K^+^ channels in nociceptive processing, particularly in determining peripheral hyperexcitability. Other pharmacologically active compounds such as diazoxide, which acts by opening potassium channels, are known to exhibit anti-nociceptive effects (Alves et al., 2004). ATP-sensitive potassium channel opening has been linked to the anti-nociception produced by systemic treatment with morphine, nonsteroidal anti-inflammatory drugs (NSAIDs), and even gabapentin (Alves et al., 2004; Tsantoulas and McMahon, 2014). ZAE therefore possibly acted through the activation of ATP-sensitive potassium channels, thus a reversal of its anti-nociceptive effect upon pretreatment with glibenclamide. This study shows that the possible involvement of the opioidergic pathway in the anti-nociceptive effect of ZAE may be linked to its interaction with ATP-sensitive potassium channels and nitric oxide (NO)-cyclic guanosine monophosphate pathways. This agrees with an earlier study that implicated the same pathways in the anti-nociceptive effect of tramadol, another important opioidergic partial agonist (Isiordia-Espinoza et al., 2014). Furthermore, nitric oxide controls ATP-sensitive potassium channels by increasing intracellular cGMP, which regulates various physiological processes including anti-nociception (Romero and Duarte, 2009).

Previous phytochemical screening of ZAE revealed the presence of alkaloids, triterpenes, flavonoids, glycosides, and steroids (Henneh et al., 2018). The presence of triterpenes was confirmed by the isolation and characterization of two triterpenes, beta-amyrin, and polpunonic acid, from the root bark of the plant (Henneh et al., 2020). β-amyrin and polpunonic acid, similar to the extract, also exhibited significant analgesic effects with comparable efficacy to morphine. This indicates that the analgesic effect of the extract could be partly or fully attributed to the presence of these compounds. As such, further development of the two compounds could lead to the discovery of potent and efficacious analgesics to complement the already existing ones.

## Conclusion

Overall, *Ziziphus abyssinica* hydroethanolic root bark extract exhibited analgesic property in this study. Our data suggest that the analgesic effect is related to its modulatory influence on opioidergic, ATP-sensitive potassium channels, and nitric oxide-cyclic GMP pathways in addition to reducing the activities of TNF-α, IL-1β prostaglandin E_2,_ and bradykinin. The analgesic effect exhibited by the extract in this study may be related to the presence of the bioactive triterpenes, β-amyrin, and polpunonic acid, isolated from the plant.

## Data Availability

The original contributions presented in the study are included in the article/supplementary material, further inquiries can be directed to the corresponding authors.
